# Adventitious rooting declines with the vegetative to reproductive switch and involves a changed auxin homeostasis

**DOI:** 10.1093/jxb/eru499

**Published:** 2014-12-24

**Authors:** Amanda Rasmussen, Seyed Abdollah Hosseini, Mohammed-Reza Hajirezaei, Uwe Druege, Danny Geelen

**Affiliations:** ^1^Plant Production, Faculty of Bioscience Engineering, Ghent University, Coupure links 653, Ghent 9000, Belgium; ^2^Plant and Crop Sciences, The University of Nottingham, Sutton Bonington LE12 5RD, UK; ^3^Leibniz Institute of Plant Genetics and Crop Plant Research, Correnstrasse 3, 06466 Gatersleben, Germany; ^4^Leibniz Institute of Vegetable and Ornamental Crops (IGZ), Kuehnhaeuser Strasse 101, 99090 Erfurt, Germany

**Keywords:** Adventitious roots, auxin, cytokinin, flowering induction, jasmonic acid, maturation, strigolactone.

## Abstract

Adventitious rooting, whereby roots form from non-root tissues, is critical to the forestry and horticultural industries that depend on propagating plants from cuttings. A major problem is that age of the tissue affects the ability of the cutting to form adventitious roots. Here, a model system has been developed using *Pisum sativum* to differentiate between different interpretations of ageing. It is shown that the decline in adventitious rooting is linked to the ontogenetic switch from vegetative to floral and is mainly attributed to the cutting base. Using *rms* mutants it is demonstrated that the decline is not a result of increased strigolactones inhibiting adventitious root formation. Monitoring endogenous levels of a range of other hormones including a range of cytokinins in the rooting zone revealed that a peak in jasmonic acid is delayed in cuttings from floral plants. Additionally, there is an early peak in indole-3-acetic acid levels 6h post excision in cuttings from vegetative plants, which is absent in cuttings from floral plants. These results were confirmed using DR5:GUS expression. Exogenous supplementation of young cuttings with either jasmonic acid or indole-3-acetic acid promoted adventitious rooting, but neither of these hormones was able to promote adventitious rooting in mature cuttings. DR5:GUS expression was observed to increase in juvenile cuttings with increasing auxin treatment but not in the mature cuttings. Therefore, it seems the vegetative to floral ontogenetic switch involves an alteration in the tissue’s auxin homeostasis that significantly reduces the indole-3-acetic acid pool and ultimately results in a decline in adventitious root formation.

## Summary

Age-related adventitious rooting decline is linked to the switch from vegetative to floral meristem identity and may be linked to changes in auxin homeostasis reducing the available free IAA.

## Introduction

Adventitious roots, such as those that form on the base of cuttings, initiate from non-root tissues including stems or petioles. Although several morphological studies have been conducted to describe the early steps in adventitious root formation through redifferentiation of cortex cells or new divisions from cambial tissues (De Klerk *et al.*, 1999; Kevers *et al.*, 1997; Rasmussen and Hunt, 2010; Rasmussen *et al.*, 2009), surprisingly little is known about the underlying processes that cause a loss of adventious root competence. Tissue age has emerged as a major factor that affects adventitious root formation which significantly hinders horticultural and forestry industries.

**Fig. 1.**
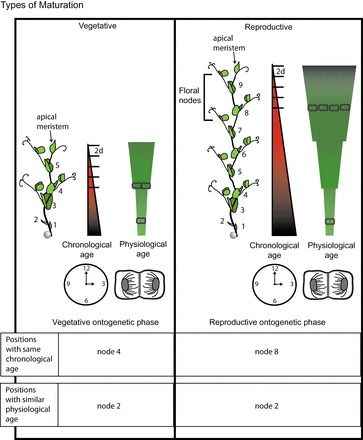
Maturation status of a plant can be described in terms of ontogenetic phase, and physiological and chronological ages. Plant on the left is in the vegetative ontogenetic phase, whereas the one on the right is reproductive with the upper nodes containing floral meristems. Numbers mark the nodes from base to apical meristem. Nodes of the same number have the same physiological age (related to cell divisions in the apical meristem at the time the tissue was laid down) in both plants whereas the chronological age (related to the passage of time since the tissue was laid down) continues to increase with time meaning node 3 on the older plant is chronologically older than node 3 on the younger plant. In addition physiological age (depicted as a more jagged progression, rather than the linear progression of chronological age) can change depending on environmental conditions and on stress responses in the plant and so is not interchangeable with inverse chronological age. In conditions described here, it takes approximately 2 d for a new leaf to emerge which marked on the chronological scale. (This figure is available in colour at *JXB* online.)

There are several ways to describe ageing in plants, which are often confounded in studies on maturation. The three main forms of maturation are illustrated in Fig. 1. The chronological age of a tissue is the time since that tissue differentiated from the meristem. As such, the newest leaves are chronologically the youngest, whereas the base of the stem is chronologically much older. Plant growth is also marked by distinct ontogenetic phases including embryonic, juvenile vegetative, adult vegetative, reproductive, and senescent (Poethig, 2003). The transition from one phase to another, and hence the chronological duration of each phase, is controlled by autonomous and environmental factors (Munne-Bosch, 2007; Poethig, 2003). The timing of phase changes, therefore, do not correspond strictly to the chronological age of a plant. Within each ontogenetic phase, the tissues differentiate from a meristem that is getting physiologically older (and has undergone more cell divisions). Physiological age may also depend on environmental conditions and stress responses of the plant and so is not the inverse of chronological age. Maturational effects can be confounded by positional effects (topophysis) which are linked to environmental effects, for example lighting effects, or specific distance effects (Leakey and Storeton-West, 1992).

Most studies investigating the decline in adventitious rooting with age do not distinguish between these three types of ageing. Instead they compare rooting in cuttings from epicotyls and hypocotyls (Diaz-Sala *et al.*, 1996) or between different locations on a single mature tree (Ballester *et al.*, 1999; Dick and Leakey, 2006; Tchoundjeu and Leakey, 1996). Each system is useful for studying adventitious root formation; however, they each pose unique difficulties when trying to discriminate between ages. Hypocotyl/epicotyl comparisons are problematic because the hypocotyl contains a pericycle-like cell layer, the founder cell layer for lateral root development, whereas the epicotyl does not (Goldfarb *et al.*, 1998). Cuttings within a single tree are confounded both by different chronological ages and topophysis resulting in exposure to different environmental conditions such as light and humidity (Matsuzaki *et al.*, 2005; Mencuccini *et al.*, 2005). Despite the valuable information provided in these studies, an understanding of how these different types of ageing regulate adventitious root formation is necessary to better understand the age-dependent decline in root initiation. To date, there has been no comprehensive study within a single species investigating adventitious root formation within and between each type of ageing.

Adventitious root formation in cuttings begins with a wound-induced increase in jasmonic acid (JA) (Schilmiller and Howe, 2005; Ahkami *et al.*, 2009), which has been linked to proliferation of adventitious roots (Ahkami *et al.*, 2009; Fattorini *et al.*, 2009). However, when present throughout the rooting period, adventitious root formation was inhibited by JA or methyl jasmonate (MeJA) in intact *Arabidopsis* hypocotyls or in *Bupleurum kaoi* cuttings (Chen *et al.*, 2007; Gutierrez *et al.*, 2012). Previous studies have not compared JA levels across different ontogenetic phases or determined how JA may correlate with the loss of adventitious root formation.

Furthermore, adventitious root induction is promoted by high auxin levels and low cytokinin levels (Bollmark and Eliasson, 1986; Bollmark *et al.*, 1988; Kuroha *et al.*, 2002) in the rooting zone whereas later stages are inhibited by auxin (Da Costa *et al.*, 2013; De Klerk *et al.*, 1999; Kevers *et al.*, 1997). Despite this clear link to adventitious root formation, auxin levels do not always correspond to rooting ability (Centeno *et al.*, 1997; Diaz-Sala *et al.*, 1996; Ford *et al.*, 2002; Osterc and Stampar, 2011). In addition, mutants with reduced cytokinin levels have increased adventitious root formation (Rasmussen *et al.*, 2012*b*; Werner *et al.*, 2003; Werner *et al.*, 2001). Cytokinins have also been linked to changes in maturation as applications of benzylaminopurine to mature material can result in expression of more juvenile characteristics (Horgan *et al.*, 1997; Mitchell *et al.*, 2004; Naik and Chand, 2003).

Recently strigolactones [a newly designated group of plant hormones found to regulate shoot bud outgrowth (Gomez-Rolden *et al.*, 2008; Umehara *et al.*, 2008)] were found to inhibit adventitious root formation (Kohlen *et al.*, 2012; Rasmussen *et al.*, 2012*b*). Inhibition of strigolactones improved adventitious root formation in a range of species (Kohlen *et al.*, 2012; Rasmussen *et al.*, 2012*a*) and opened the possibility that this hormone may be part of the decline in this process with age.

Here, a cutting system using pea was developed to separate each type of ageing. Pea mutants exist for strigolactone biosynthesis [*ramosus* (*rms*)*5*] and signalling (*rms4*) (Gomez-Roldan *et al*., 2008; Umehara *et al.*, 2008) and mutants also exist for flowering time [*die neutralis* (*dne*) and *late flowering* (*lf)*] (Foucher *et al.*, 2003; Liew *et al.*, 2009). The adventitious root phenotype has not previously been measured in the early flowering mutants so little is known regarding the role of these pathways and their early switch to reproductive ontogeny on adventitious root formation. Here it is demonstrated that the decline in adventitious root formation with age was not regulated by strigolactones and was correlated with the ontogenetic switch to reproduction and not the chronological age of the cutting base. This was supported by the reduced adventitious root formation in early flowering mutants. Measurements of endogenous hormone levels revealed that the younger cuttings had an early peak in indole-3-acetic acid (IAA) which combined with low cytokinins, resulted in a large early peak in the IAA:CK ratio. These peaks were not present in the older cuttings. In addition, the older cuttings had a delay in accumulation of JA in the cutting base. Responses of root formation and of DR5:GUS expression in juvenile versus mature cuttings to IAA application further supported an important role of auxin homeostasis in the ontogenetically controlled decline of adventitious root formation.

## Materials and methods

### Pea seedling growth/germination

Seeds of pea (*Pisum sativum*) were planted in tubes (500ml) filled with premium blend potting mix (7:2:1 pine bark:peat blend:sand) without fertilizer and watered every second day under 16h day length with day/night temperatures of 21 °C/18 °C until cuttings were taken. To test adventitious root formation in differently aged plants, seeds were planted at different dates so that the cuttings could all be taken on the same day. Lines used were torsdag wild type, *rms4-1* (strigolactone insensitive) and *rms5-3* (strigolactone deficient), *le-3* (Torsdag gibberellin deficient mutant), *dne* and *lf* (in the *le-3* Torsdag cultivar [provided by J. Weller, University of Tasmania, Australia)], and W6 22593 wild type and DR5:GUS lines (provided by P. Polowick, NRC-CNRC, Canada).

### Floral induction

Every second day starting at 7 d after germination 4 pea plants of wild type and strigolactone mutants were moved from long day (16/8h d/n) into short day (8/16 h d/n). The node of floral induction was recorded with node numbers beginning with the first scale leaf at the basal region of the stem and increasing with each subsequent node to the apical meristem (see Fig. 1). This was repeated in two independent experiments.

### Pea cutting conditions

An adventitious root formation system was established using peas of different ages and from which cuttings could be taken at different positions along the stem (Supplementary Table S1). Plants were grown for 10, 14, 18, 22, or 26 d to cover vegetative and floral ontogenetic developmental stages. Cuttings were taken above the second scale leaf such that the bases had different chronological ages and the apex had different physiological ages. To control for different size cuttings a second set of cuttings were decapitated leaving all the cuttings with 2 nodes. A third set of cuttings consisted of only the upper two nodes, in which the bases were the same chronological ages and the apical meristems were different physiological ages. Cuttings from node 2 (just above the second scale leaf; see Fig. 1 for numbering) taken at 14 d after germination typically had one extra node than decapitated cuttings or cuttings of the top two nodes. The total number of leaves was recorded at the end of each experiment. Cuttings of the dwarf early flowering mutants had the lower leaf (in 14 d after germination) or lower two leaves (in 22 d after germination) removed.

In all cases bases were placed in 20ml tap water, shoots were placed in the light and cutting bases in the dark. The water was replaced as required. After 21 d, the rooting percentage (percentage of all the cuttings on which roots formed), the average number of adventitious roots per cutting (including every cutting planted, even those which did not form adventitious roots), average number of leaves expanded, and the level of flowering (no flowers, flower buds, open flowers, seeds present) were recorded.

### Hormone treatments

To test responsiveness to JA (0, 0.1, 1 µM), or IAA (0, 0.3, 1, 3 µM), treatments were applied to the cut bases of juvenile or mature cuttings for the first 6h and were then replaced by water. 10 µl of GA (0, 1 or 10 µM) was applied to the apical meristems of wild-type or *le* (GA-deficient) pea seedlings 4 d before cutting. The treated plants all continued to exhibit enhanced stem elongation throughout the experiment with the *le* dwarf plants growing to the same length as the wild type.

### DR5:GUS expression

GUS staining of pea DR5::GUS cuttings (DeMason and Polowick, 2009) was performed as described by Vanneste *et al.* (2005). Staining was left for 24h before transferring to water. Cross sections were cut by hand and mounted in water on slides before viewing.

### Grafting

Torsdag wild type plants were grown as described above for 12 and 22 d. Grafts were performed with the upper node grafted to plants decapitated in the internode above the most recently expanded leaf. After 13 additional days, cuttings were taken at node 2 (as in Fig. 3A which was always within the rootstock tissue) and the number of adventitious roots quantified. The juvenile root stocks contained 2 nodes, whereas mature root stock contained 10 nodes. Final scion/root stock combinations included: old/old, old/young, young/old, and young/young (Supplementary Fig. S1). Once grafting was complete every plant had an intact apical meristem. The plants were covered with clear plastic to reduce water loss. After 4 d the bags were partially opened and after a further 4 d the bags were removed. The experiment was also repeated using the cultivar W6 22593 plants grown for 8 and 24 d before grafting. In this experiment leaves were removed at the time of grafting but it was otherwise the same as described above.

### Hormone sampling

Plants were grown as described above for 12 or 30 d. Cuttings were taken above the second scale leaf and were different sizes (Supplementary Table S1). Samples of the rooting zone region of the cutting bases (12–15mm from the base) were taken immediately (intact tissue), after 30min, and 6, 24, and 96h, snap frozen in liquid nitrogen and stored at –80 °C. Sixteen cuttings of each age were kept to test the adventitious root phenotype after 21 d.

### Hormone analysis

For the analysis of indole-3-acetic acid (IAA) and jasmonic acid (JA) concentration by GC-MS/MS, the extraction and clean-up of plant extracts were carried out according to Ahkami *et al.* (2013) based on the multiplex GC-MS/MS technique described by Müller *et al*. (2002). Approximately 150mg FW of shock-frozen tissue was extracted using (^2^H)_2_–IAA and (^2^H)_6_–JA as internal standards. General (and IAA-specific) GC and MS settings are described by Ahkami *et al.* (2013). MS settings for endogenous JA were: parent ion=225 (M+H)^+^, diagnostic product ion=207, excitation amplitude 0.5V. A second channel analysing the isotopically labelled standard (^2^H)_6_–JA used the parent ion (*m*/*z*)=231 (M+H)^+^ and the diagnostic daughter ions (*m*/*z*)=210+211+212+213. The amount of endogenous compound was calculated from the signal ratio of the unlabelled over the corresponding stable isotope-containing mass fragments. When the signals of JA were below the limit of quantification (LOQ) of 1.5 pmol per injection (=10 pmol g^–1^ FM), the values were set to LOQ/2.

Analyses of cytokinins (CKs) concentration by UPLC-ESI-MS/MS. All UPLC-ESI-MS/MS experiments were carried out using an Agilent 1290 infinity system connected to an Agilent triple quadruple mass spectrometer QQQ6490 (Agilent Germany). Separated compounds were ionized at atmospheric pressure via electrospray and directed to the mass spectrometer. The control of the complete system and recording of the spectra were performed with the MassHunter, release B.04.00 (B4038).

UPLC-ESI-MS/MS conditions: To separate the individual CKs a UPLC system was used including a gradient pump, an autosampler, and a column compartment. Separation was carried out using a high-capacity column (Eclipse Plus C18, RRHD 1.8 µm, 2.1×50mm). Gradient was accomplished with LCMS grade water (Chem solute, Th. Geyer, Germany) containing 0.1% formic acid (Fluka, Germany) as buffer A and LCMS grade methanol (Chem solute, Th. Geyer, Germany) including 0.1% formic acid as buffer B. The column was equilibrated either with a mixture of buffer A (86.5%) and buffer B (13.5%) at a flow rate of 0.4ml min^–1^ and heated at 40 °C during the whole measurement. The gradient was produced by changes of the buffer B as follows: 0–5min at 18%, 5–6min at 70%, 6–7min at 99%, 7–7.1min at 13.5%, and kept up to 9min at 13.5%. The whole duration of the run was 9.0min.

MS/MS analysis was performed using a triple quadruple 6490 of the Agilent Company. The following parameters were employed: desolvation temperature 350 °C, desolvation nitrogen gas of 720 l hr^–1^ for both, capillary voltage 2.0 KV, detection in positive ion mode and different dwell times between 20 and 200 s. Collision energy differed among the compounds (Supplementary Table S2). Protonated ions [M–H]^+^ were monitored with a span of 1 amu. Multiple reactions monitoring (MRM) was performed to identify individual compounds accurately. This allows minimizing parallel monitoring and enhancing the sensitivity. As internal standards and for testing the stability of the instruments and the retention times, either [2H6–ABA] or a mixture of [15N4]–*cis*-zeatin, [2H5]–*trans*-zeatinriboside were used.

## Results

### Time of floral meristem establishment

**Fig. 2.**
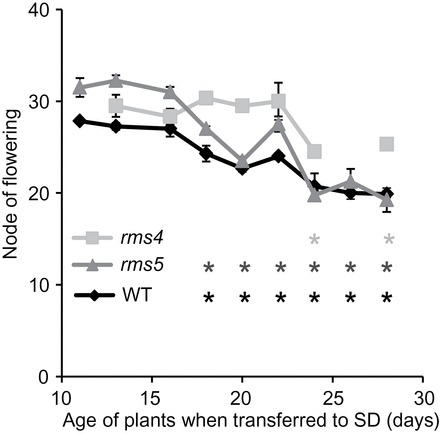
Irreversible floral induction is delayed in *rms4*. Wild type (WT) and *rms5* (strigolactone deficient) switch to floral meristems earlier than *rms4* (strigolactone insensitive). Significance values (*, *P*<0.05, students *t-test*) compare the node of flowering of each consecutive time point to the earliest transfer within each genotype (11 or 13 d after germination). Light grey, *rms4*; dark grey, *rms5*; black, wild type. Means are presented ± standard error. *n*>8.

It is important to know which ontogenetic phase the plants are in when cuttings are harvested so experiments can be designed for vegetative-only, reproductive-only, or to cross the vegetative–reproductive boundary. Meristems become determined for floral development well before the flowers are visible making it difficult to see what ontogenetic phase the plants are in. Once determined, the node of flowering will no longer change and this can be exploited using day length as peas flower later in short days so produce flowers at a higher node than those grown in long days. Figure 2 shows the node of flowering when plants are grown in long days (where the node of flowering will be lower and hence a lower node number as shown in Fig. 1) and then transferred to short days (where the node of flowering will be higher). Once the nodes have become determined for flowering in the long-day conditions this will no longer change when transferred to short-day conditions, telling the time when the plants switch into the reproductive phase. This switch has not previously been tested for the strigolactone mutants. In wild type and in *rms5* the switch to flowering began 18 d after germination (Fig. 2) as seen by the significant difference in the node of flowering at this transfer time (illustrated by the first black and light grey asterisks in Fig. 2) compared with the node of flowering at the earliest transfer time (13 and 11 d after germination for *rms4* and wild type, respectively). This was comparable to previously published results with *le* in the Torsdag background (Hecht *et al.*, 2011). However, *rms4* had a delayed flowering phenotype with the irreversible transition beginning 22 d after germination (as shown by the first asterisk in dark grey under the graph in Fig. 2). Although the regulation of this delay is not the focus of this study, the delayed transition to flowering is of importance for differentiating between ontogenetic phase and other forms of ageing on adventitious root formation in subsequent experiments.

### Adventitious rooting declines after the switch to floral meristem identity independently of strigolactone

**Fig. 3.**
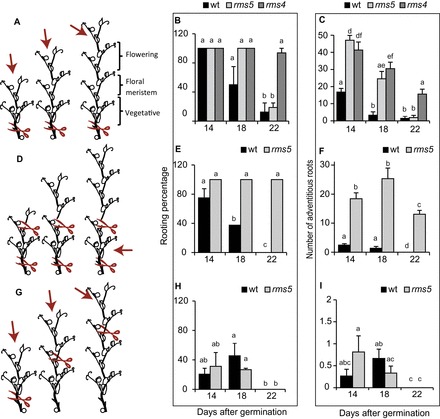
Cuttings can be taken at different chronological ages, or different physiological ages, within or across different ontogenetic ages. (A, D, G) Cartoon representations showing the location of cutting (red scissors) and which section of the stem was used for the adventitious rooting assay (red arrows); however the number of nodes in the older cuttings are not necessarily representative as this changes slightly with genotype (Supplementary Fig. S6). (A) Cuttings taken above node two have different chronological age at the cutting base, different physiological age of the apical meristem, and different size cuttings crossing the vegetative–floral ontogenetic switch. (D) Cuttings taken above node 2 but decapitated have different chronological age at the cutting base, different physiological age of the original apical meristem, and have the same size cuttings crossing the vegetative–floral ontogenetic switch. (G) Cuttings taken with two leaves expanded have bases with the same chronological age, apical bud with different physiological age, and same size cuttings crossing the vegetative–floral ontogenetic switch (Supplementary Table S1). (B, E, H) Changes in rooting percentage with age and (C, F, I) adventitious root formation declines in cuttings taken as illustrated in A, C, and E respectively. Means are presented ± standard error; different letters represent significantly different means (Students *t*-test). *n*>16. Rooting percentage was calculated per rooting box and then averaged to produce the standard error bars and statistics. Where bars are missing the standard error was 0. (This figure is available in colour at *JXB* online.)

As strigolactones have been identified as inhibitors of adventitious root formation (Kohlen *et al.*, 2012; Rasmussen *et al.*, 2012*b*), the strigolactone-deficient and -insensitive mutants of pea were used to determine if adventitious root formation remains high in these mutants with increasing age compared with the wild type. Contrary to this hypothesis, the wild type, strigolactone-deficient (*rms5*) and -insensitive (*rms4*) mutants all produced less adventitious roots in the older cuttings (Fig. 3C, F, and I; Supplementary Fig. S2) demonstrating that strigolactone is not responsible for the decline in adventitious root formation with age.

During the vegetative phase the rooting percentage was unaffected by chronological or physiological age at the cutting location (Supplementary Fig. S2A). The number of adventitious roots formed increased with age in all genotypes from 10 d old plants to 18 d old plants (Supplementary Fig. S2B). Wild type increased 3.75-fold from 8 roots per cutting in 10-d-old plants to 30 roots per cutting in 18-d-old plants. *rms4* and *rms5* increased 2.5- and 2-fold, respectively, from 21 roots per cutting in each to 52 roots per *rms4* cutting and to 41 roots per *rms5* cutting from 18-d-old plants.

After the switch to reproductive ontogenetic phase, cuttings taken above node 2 from 30-d-old wild-type plants had a rooting percentage of 6% (Supplementary Fig. S3A) and the average number of adventitious roots was 0.13 (Supplementary Fig. S3B). In the days following the transition to reproductive phase, rooting percentage of decapitated cuttings was slightly reduced in wild type (26 d after germination), but were unaffected in the other ages or in *rms5* (Supplementary Fig. S4A), whereas there was a steady decline in adventitious root formation from 15 to 5 roots in wild type and from 20 to 11 in *rms5* (Supplementary Fig. S4B).

The above findings suggest that the reduction in adventitious root formation is linked to the transition to flowering. To test this, cuttings were taken before, during, and after the vegetative–reproductive switch. Cuttings from the same position (node 2) but of different sizes showed a reduction in rooting percentage from 100 to 50 to 10 for 14, 18 and 22 d after germination, respectively, in wild type, and from 100% for 14 and 18 d after germination to 10% at 22 d after germination in *rms5* corresponding to crossing the floral transition (Fig. 3B). This was also observed in decapitated wild-type cuttings (same size) with a drop from 75% to 0, whereas *rms4* was unaffected in both cases (Fig. 3E). The number of adventitious roots also declined after the transition to reproductive ontogenetic phase in wild type and strigolactone mutants (from 17, 47, and 41 roots at 14 d after germination to 1.5, 1.9, and 16 in wild type, *rms5*, and *rms4*, respectively; Fig. 3C). The number of adventitious roots was unchanged at 18 d after germination but dropped in decapitated cuttings from 2.5 and 18 in wild type and *rms5* at 14 d after germination to 0, and 13 in wild type and *rms5* respectively at 22 d after germination (Fig. 3F). These findings suggest that the size of the cutting is not the main cause of the decline in adventitious root formation. Decapitating did, however, reduce the number of adventitious roots in all cuttings compared with intact cuttings (Fig. 3C compared with Fig. 3F).

When cuttings were taken at different positions (and so the base had the same chronological age but different physiological ages), the rooting percentage was reduced from 20 and 31% in wild type and *rms5* to 0% in both (Fig. 3H). The number of adventitious roots followed a similar trend to rooting percentage with a decline with age although this was not significantly different for wild type (Fig. 3I). The reduction in rooting percentage and number of adventitious roots taken from higher positions along the stem compared with cuttings harvested from node 2 supports other studies suggesting that adventitious root formation inversely correlates to physiological age of the cutting location. However, as discussed earlier, cuttings collected lower on the stem with visible flowers still produced no adventitious roots suggesting that ontogenetic phase has a greater influence over adventitious rooting than physiological age.

### Flowering mutants

**Fig. 4.**
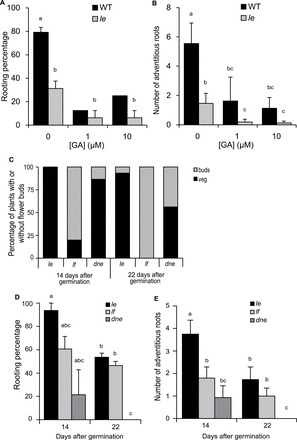
GA deficient (*le*) cuttings produce less adventitious roots than wild type. Early flowering (*dne* and *lf*) cuttings produced less adventitious roots. WT, wild type torsdag; *le*, *le-3* NGB5839 torsdag controls; *lf*, *late flowering*; *dne*, *die neutralis* early flowering; *lf* and *dne* are in *le* torsdag background. Rooting percentage (A) and number of adventitious roots (B) in wild type and *le* with increasing doses of GA3 applied to the apical meristem before cutting; (C) percentage of cuttings with floral buds (buds) or not (veg); (D) rooting percentage, and number of adventitious roots (E) in cuttings taken from the early flowering mutants at 14 and 22 d after germination compared with the *le* control. Means are presented ± standard errors. Different letters represent significantly different means (Students *t-test*). *n*>16. Rooting percentage was calculated per rooting box and then averaged to produce the standard error bars and statistics. Where bars are missing the standard error was 0.

To confirm a role of flower transition in adventitious rooting capacity, the early flowering pea mutants *dne* and *lf* were used, which are in the *le-3* (NGB5839) gibberellin deficient background of Torsdag. Figure 4A shows that gibberellin deficiency inhibits adventitious rooting in pea as the *le-3* mutant produced fewer adventitious roots compared with the wild type. However, when GA was applied to cuttings of wild type or *le-3* both the rooting percentage and number of adventitious roots was inhibited (Fig. 4A and 4B) suggesting that either a minimal level of GA is required for adventitious rooting and higher levels inhibit, or suggesting the *le-3* mutant secondarily affects other signalling networks which control adventitious rooting. To compare the relative level of flower induction, the numbers of cuttings with floral buds present (buds) or absent (veg) were recorded for the cuttings at the time of scoring (Fig. 4C). The *lf* and *dne* mutants both already had floral buds (80% and 13% of cuttings, respectively) in cuttings taken 14 d after germination, whereas the *le-3* controls had none. By 22 d after germination 100% of the *lf*, 44% of *dne*, and only 7% of *le-3* cuttings had floral buds (Fig. 4C). This supports the published early flowering phenotype of *dne* and *lf* and also supports that the *le-3* mutant is unaffected in flowering transition (Hecht *et al.*, 2011).

Using cuttings taken from above node 2 (as in Fig. 3A), the rooting percentage was reduced in cuttings from plants 22 d after germination compared with those 14 d after germination. The rooting percentage was lowest in cuttings from the *dne* mutant and *lf* was least affected by age (Fig. 4D). Similarly there was a decline in the number of adventitious roots from 14 to 22 d after germination, which also crossed the floral transition in the *le-3* background (Fig. 4E). Both of the early flowering mutants produced less adventitious roots already at 14 d after germination compared with the *le-3* controls. All genotypes produced less adventitious roots at 22 d after germination with *dne* failing to form any at this age. These findings support the previous studies that revealed a link between adventitious root formation and the switch from vegetative to reproductive ontogenetic phases.

### The flowering-related inhibition signal is present in the base of cuttings

**Fig. 5.**
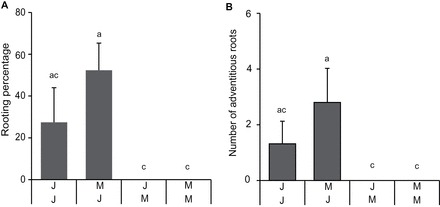
Grafting combinations demonstrate that the rooting percentage is dependent on the age of the root stock and hence base of the cutting. J/J are juvenile shoots grafted onto juvenile roots; M/J are mature shoots grafted onto juvenile roots; J/M are juvenile shoots grafted onto mature roots; M/M are mature shoots grafted onto mature roots (Supplementary Fig. S1). (A) Rooting percentage. (B) Number of adventitious roots. (A, B) Means are presented ± standard error; *n*=16. Different letters represent significantly different means (Students *t-test*).

In the above sections it was determined that strigolactone is not the cause for the loss in adventitious rooting ability and suggest that the signal is linked to the switch to reproductive ontogeny. However, it is unknown from where the signal originates and whether the signal is mobile or restricted to the base of the cuttings. Therefore, reciprocal grafting experiments were conducted (Supplementary Fig. S1) using wild-type Torsdag plants grown for 12 and 22 d (Fig. 5) and in W6 22593 grown for 8 and 24 d (Supplementary Fig. S5) before grafting.

If the inhibitory signal was produced in the shoot apex and transported to the base it would be expected that graft combinations with mature scions to have significantly reduced root formation compared with graft combinations with juvenile scions. Instead, it was observed that the rooting percentage was reduced to 0 in Torsdag (Fig. 5A) or by 20% in W6 22593 (Supplementary Fig. S5A) in the two graft combination with mature bases. The number of adventitious roots formed was not significantly affected by the different graft combinations in W6 22593 (Supplementary Fig. S5B), whereas in Torsdag (Fig. 5B) the number of adventitious roots on cuttings with a mature base was zero and significantly lower than cuttings taken from the mature/juvenile combination. These findings suggest that the rooting percentage is dependent on the physiological status of the base of the cutting and less on that of the apical meristem.

### The decline in adventitious root formation with age is not related to the number of leaves expanded

To determine the effect of leaf number on adventitious root formation, the total number of leaves on each cutting were measured. Cuttings taken from node 2 had more leaves expanded with increasing age (Supplementary Fig. S6A), whereas the rooting declined (Fig. 3). Cuttings of *rms5* and *rms4* strigolactone mutants, which produced more adventitious roots, had more leaves expanded compared with wild type (Supplementary Fig. S6A). Also the early flowering mutants have a decline in both root formation and leaf number compared with the *le* wild-type control (Supplementary Fig. S6B). In the other sets of cuttings the number of leaves was deliberately controlled with either decapitation (Supplementary Fig. S6C) or by using the top two nodes (Supplementary Fig. S6D) and in these cuttings the decline in adventitious root formation with age was conserved.

For the grafting experiments the number of leaves expanded above the graft were also recorded (this is the number of leaves grown during the rooting period as all existing leaves were removed at the time of cutting). The number of leaves grown was highest in graft combinations with juvenile scions and mature bases for Torsdag (Supplementary Fig. S6E) and for W6 22593 were reduced the most in the combination with mature scion and juvenile base (Supplementary Fig. S6F). These findings demonstrate that the decline in adventitious root formation with age is not tightly linked to the number of leaves expanded.

### Hormone analysis and response of pea cutting bases at different ages

**Fig. 6.**
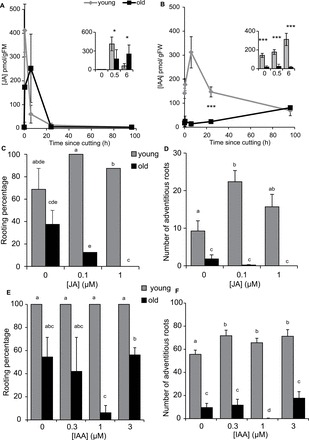
Endogenous levels of jasmonic acid and auxin in the rooting zone, and the exogenous response to jasmonic acid and auxin in the rooting zone. (A, B) Endogenous levels of jasmonic acid (JA) and auxin (IAA) in the rooting zone. (C–F) Exogenous response to JA (C, D), and IAA (E, F) in the rooting zone. JA levels at 0 and 96h were generally below the LOQ of 10 pmol g^–1^ FM and were set to LOQ/2 (5 pmol g^–1^ FM). The same was applied to 2 and 4 individual samples of young and old cuttings at the 24h time point, respectively. Concentrations of JA (A) and IAA (B; pmol per gram fresh weight). Subset panels (A, B) are the first three time points (0, 0.5, and 6h) for each of the respective full time lines. Rooting percentage (C, E) and number of adventitious roots (D, F) in young and old cuttings with increasing doses of JA (C, D) or IAA (E, F); means are presented ± standard errors. (A, B) Significantly different means *, *P* <0.05; ***, *P* <0.001; Welch two sample *t*-test. (C–F) Different letters represent significantly different means (Students *t-test*). *n*=16 for treatments. *n*=5 for hormone analysis. Rooting percentage was calculated per rooting box and then averaged to produce the standard error bars and statistics. Where bars are missing in panel D this was because only one rooting box was used and where error bars are missing in panel F the standard error was 0.

Given the observation that the physiological status of the grafted base determined rooting ability, the concentrations of jasmonic acid (Fig. 6A), auxin (Fig. 6B), and different cytokinins in the bases of cuttings taken above node 2 (similar to Fig. 3A) were measured. Sixteen cuttings of each age (12 or 30 d after germination) were allowed to root and as shown throughout this study the rooting percentage and the number of adventitious roots were inhibited in mature cuttings (Supplementary Fig. S3A and B). Juvenile cuttings had no floral buds at the time of scoring, whereas 6% of the cuttings from mature cuttings had formed seeds, 6% had flowers, 56% had unopened floral buds, and only 31% were still vegetative (Supplementary Fig. S3C). These findings demonstrate the clear difference in rooting ability of the two sets of samples from which hormone analysis was conducted.

#### Jasmonic acid:

JA is a wound response hormone that is induced very quickly after wounding. By 30min after cutting, JA levels had increased by more than 40-fold in the juvenile cuttings and had dropped to background levels by 6h (Fig. 6A). JA production in the older cuttings peaked later (at 6h) before returning to background levels (20h). In addition, the later peak in JA seems to be lower than for the younger cuttings, although this was not significant. These findings suggest that the older cuttings were slower in responding to wounding compared with the younger cuttings.

JA was applied to pea cuttings, which increased adventitious root formation in the juvenile cuttings with a maximum induction of both rooting percentage (Fig. 6C) and number (Fig. 6D) at 0.1 µM. In contrast, JA seems to further inhibit adventitious root formation in the mature cuttings, although this trend was not significant (Fig. 6D).

#### Auxin:

Auxin is a well-known inducer of adventitious root formation. The IAA concentration in the cutting bases of the younger peas (bases at time 0) was much higher than that in the older plants (Fig. 6B). IAA then peaked at 6h after cutting in the younger tissues and decreased to background levels by 24h and finally dropped further by 96h after cutting (Fig. 6B). This early IAA peak was not seen in the older cuttings, suggesting auxin levels do not increase as much as in younger cuttings, and that the peak is much later in the older cuttings. To determine if exogenous IAA could rescue adventitious root formation in mature cuttings, increasing doses of IAA (0, 0.3, 1, 3 µM) were added for the first 6h after cutting. All three doses enhanced the number of adventitious roots in juvenile cuttings (Fig. 6F) but were unable to promote adventitious root number (Fig. 6F) or percentage (Fig. 6E) in mature cuttings.

**Fig. 7.**
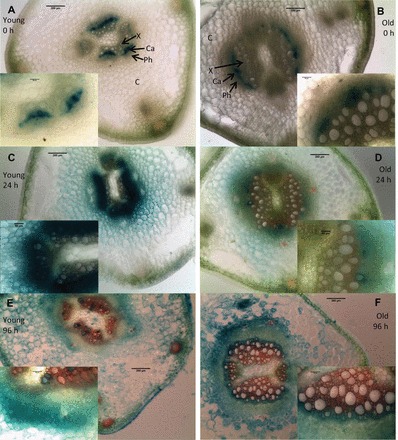
DR5:GUS expression in the bases of young (A, C, E) or old (B, D, F) cuttings stained at the time of cutting (A, B), 24h after cutting (C, D), or 96h after cutting (E, F). C, cortex; Ph, phloem; Ca, cambium; X, xylem. Samples were cleared for 45min in acetone on ice, stained in x-gluc for 24h, free hand sections made and photographed mounted in water. Subset panels from same images at higher cellular resolution. Sections were cut from four separate stems for each age/time combination. Inset images are of the vascular bundles (containing xylem, cambium, and phloem). Bars in full size panels are 200 µm, bars in the inset panels are 60 µm.

**Fig. 8.**
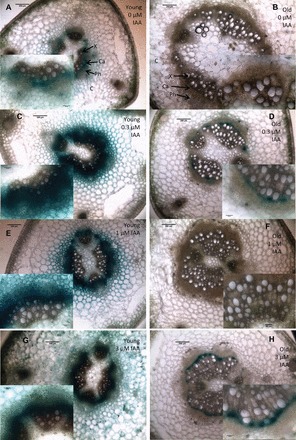
DR5:GUS expression in the bases of young (A, C, E, G) or old (B, D, F, H) cuttings treated with 0 µM (A, B), 0.3 µM IAA (C, D), 1 µM IAA (E, F), or 3 µM IAA (G, H) and all stained at 24h after cutting. C, cortex; Ph, phloem; Ca, cambium; X, xylem. Samples were cleared for 45min in acetone on ice, stained in x-gluc for 24h, free hand sections made and photographed mounted in water. Subset panels from same images at higher cellular resolution. Sections were cut from four separate stems for each age/treatment combination. Inset images are of the vascular bundles (containing xylem, cambium, and phloem). Bars in full size panels are 200 µm, bars in the inset panels are 60 µm.

DR5:GUS expression also demonstrated a strong increase in auxin response at 24h after cutting in juvenile bases (Fig. 7A, C, E), whereas the biggest increase in mature bases occurred at 96h after cutting (Fig. 7B, D, F). Juvenile bases (Fig. 7A, C, E) always had stronger DR5:GUS expression compared with mature bases (Fig. 7B, D, F). DR5:GUS expression was also highly auxin inducible in young cutting bases when observed 24h after cutting (Fig. 8A, C, E, G). However, in mature bases (Fig. 8B, D, F, H) with the same treatments only the highest auxin treatment (Fig. 8H) had any visible effect on DR5:GUS expression and this level is still lower than in the juvenile bases. This strongly indicates that in cutting bases of mature pea the arriving IAA is rapidly inactivated so that the IAA signal cannot be transduced into induction of adventitious roots.

#### Cytokinins:

**Fig. 9.**
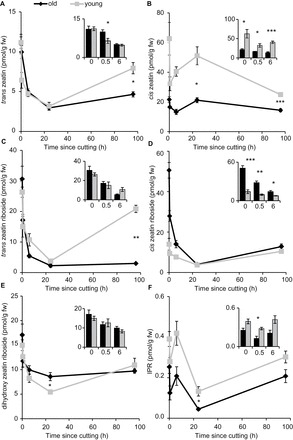
Cytokinin concentrations follow similar trends in the rooting zone of old and young cuttings. (A) *trans*-zeatin, (B) *cis*-zeatin, (C) *trans*-zeatin riboside, (D) *cis*-zeatin riboside, (E) dihydroxyzeatin riboside and (F) isopentenyl riboside (IPR) at 0, 0.5, 6 (also in each subset bar graph), 24, and 96h after cutting. Means presented ± standard error bars. *n*=5. Significantly different means *, *P*<0.05; **, *P*<0.01; ***, *P*<0.001 Welch two sample *t*-test.

Cytokinins (CKs) have an overall inhibitory effect on adventitious root induction, but can have a promotive effect during the first 24h, when CKs start to drive cell cycle movement (Da Costa *et al.*, 2013). Levels of different CKs are also linked to flowering transition (Valdes *et al.*, 2002; Valdes *et al.*, 2004*a*; Valdes *et al.*, 2003). The levels of CKs were therefore measured in the bases of the cuttings from the young (12 d after germination) and mature (30 d after germination) pea plants. The different cytokinins measured were *trans*-zeatin, *cis*-zeatin, *trans*-zeatin riboside, *cis*-zeatin riboside, dihydroxyzeatin riboside, and isopentenyl riboside (IPR; Fig. 9A–F). In all cases there was an initial reduction in CK concentration in the bases of both old and young cuttings, followed by an increase at later times. In the case of *cis*-zeatin there was an immediate decrease which was rescued by 24h and then decreased again (Fig. 9B). IPR concentrations in both old and young tissues decreased within 0.5h, were rescued by 6h, and decreased further by 24h before recovery by 96h (Fig. 9F).

*Trans*-zeatin (Fig. 9A) and *trans*-zeatin riboside (Fig. 9C) levels were similar between young and old cutting bases except at 96h when the young bases contained higher levels than the older cutting bases. Dihydroxyzeatin riboside concentrations were similar in old and young tissues except at 24h when the concentration in the younger tissue reduced further than for the older tissue (5 compared with 9 pmol g^–1^ FW, respectively, Fig. 9E).

There was less *cis*-zeatin (Fig. 9B) and IPR (22 and 0.25 pmol g^–1^ FW respectively; Fig. 9F) in the old tissue compared with the young (63 and 0.39 pmol g^–1^ FW respectively). For both of these cytokinins there was more in the young compared with the old throughout the 96h of sampling. At time 0 the main difference between young and old cuttings was that there was 50.6 pmol g^–1^ FW *cis*-zeatin riboside in the old cuttings compared with 13 pmol g^–1^ FW in the young bases. This trend holds until 24h post cutting, after which there was no difference between old and young tissue (Fig. 9D).

These results show that trends in cytokinin concentration dynamics are similar in both old and young tissues, however the levels of some CKs do vary at later time points between the rooting and non-rooting cuttings.

#### Auxin to cytokinin ratios:

It has been suggested that the ratio of auxin to cytokinin is more important for adventitious root formation rather than the individual hormone concentrations (Blakesley, 1994; Ludwig-Muller, 2009). The ratios of IAA to *trans*-zeatin, *cis*-zeatin, *trans*+*cis*-zeatin and to total CKs were therefore calculated (Supplementary Fig. S7). For IAA:*trans*-zeatin, there was a large increase in the young cutting bases from 12 to 66 by 6h, whereas there was no change in the bases of old cuttings staying around 1.5 (Supplementary Fig. S7A). After 6h the peak in young cuttings gradually returned to T0 levels, whereas in the bases of old cuttings the ratio gradually increased to 96h ending at 18h. For IAA:*cis*-zeatin (Supplementary Fig. S7B) and for IAA:*trans*+*cis*-zeatin (Supplementary Fig. S7C) the pattern was similar with a peak in the young cutting bases at 6h, which had almost recovered by 24h. In the older cuttings the increase from 24 to 96h was steeper than for the IAA:*trans*-zeatin ratio. The ratios for IAA:*cis*-zeatin were all lower than for *trans*-zeatin because the levels of *cis*-zeatin were higher. The same trend is followed for IAA:total CK (Supplementary Fig. S7D) with a peak in the young cuttings and a late rise in the older cuttings. These findings support the possibility that the reduction in adventitious rooting in cuttings from older plants may be due to the loss in the IAA:CK peak at 6h after excision.

## Discussion

### Floral phase change responsible for adventitious rooting decline

In this study an experimental system has been developed for separating the different types of ageing and their effects on adventitious root formation, and shown that the loss of adventitious root formation is correlated with the ontogenetic switch from vegetative to floral meristems. Previous studies have found that adventitious roots declined as flowers developed on stock plants of chrysanthemum (DeVier and Geneve, 1997). However, buds in leaf axils are known to switch to floral long before flowers are visible (Taylor *et al.*, 2002). DeVier and Geneve (1997) suggested that flower buds compete for resources, but in this study adventitious root inhibition occurred when meristems switch from vegetative to floral but before flower appearance. This suggests that the root-related decline in adventitious rooting is linked to the signals controlling the switch to flowering or very soon after and not to a signal or resource competition produced by the flowers themselves.

### Strigolactones are not responsible for the decline in adventitious root formation with age

Mutants defective in strigolactone production (*rms5*) and mutants insensitive to strigolactones (*rms4*) both displayed the decreased adventitious root formation seen in the wild type with the switch to flowering, demonstrating that this phenotype is not a result of increased strigolactone production or signalling. In some situations the strigolactone mutants were slightly less sensitive to the decline in age possibly owing to an overall increase in auxin transport (Shinohara *et al.*, 2013), which may partially overcome the reduction in the early auxin peak in the older plants. In *rms4* this may also be linked to the delay in flowering induction described below. Either way, the response of the strigolactone mutants to the floral transition suggests that strigolactones regulate adventitious root formation primarily in vegetative cuttings.

Previously, it has been shown that *max2* has a delayed senescence phenotype (Shen *et al.*, 2007; Woo *et al.*, 2001) and in petunia the strigolactone mutants also have delayed flowering (Napoli, 1996; Snowden *et al.*, 2005). Other *MAX2/RMS4* specific phenotypes have also been reported for response to light (Shen *et al*., 2007, 2012), karrikins (Nelson *et al.*, 2011), nodulation (Foo *et al.*, 2013), and light-grown hypocotyl elongation (Shen *et al.*, 2012; Stirnberg *et al.*, 2002). These studies are consistent with the findings that *rms4* has a delayed flowering phenotype.

### The jasmonic acid peak is reduced and delayed in mature cuttings

Wounding is an intrinsic factor in excision of cuttings and in the majority of plants wounding leads to an increase of jasmonates (Schilmiller and Howe, 2005). JA promoted adventitious root formation in juvenile pea cuttings (Fig. 6C) and in *Arabidopsis* thin cell layers (TCLs) (Fattorini *et al.*, 2009). The induction of adventitious roots in pea cuttings by JA is opposite to the findings in etiolated *Arabidopsis* hypocotyls where impaired JA conjugation or exogenous treatment with JA inhibited adventitious root formation (Chen *et al.*, 2007; Gutierrez *et al.*, 2012). By contrast, the older pea cuttings showed a later accumulation of JA, but also did not respond to JA treatments supporting the suggestion that the later JA peak is not the only factor affecting the reduced adventitious rooting with age

It is also possible that the intact hypocotyl system may have different requirements of JA for adventitious root initiation compared with the wounding-related pea cuttings or *Arabidopsis* TCLs. Considering these relationships, the postponed accumulation of JA in the older cuttings may have impaired adventitious root formation by delaying the sink establishment (Ahkami *et al.*, 2009) and by inhibiting initiation of root development thereafter.

### Changes in auxin metabolism linked to decline in adventitious root formation with age

Adventitious root formation in petunia cuttings was induced by an early peak of IAA in the cutting base independent of polar auxin transport (Ahkami *et al.*, 2013). In the present study with pea, the lower IAA level at the time of cutting, the missing IAA peak after six hours (Fig. 6B), and the lack of DR5:GUS response to IAA application in the mature when compared with the juvenile cuttings (Fig. 7) suggest a strong inactivation of arriving IAA in the base of mature cuttings. Combined with the lower rooting capacity of these same cuttings (Fig. 3C), and the missing auxin response of adventitious root formation (Fig. 6E, F), auxin inactivation may be part of the reason for the decline in adventitious root formation with age. Osterc and Stampar (2011) combined differently aged cuttings of *Prunus* with IBA applications and blocking of phloem transport by girdling when studying the temporal course of IAA, IBA, and IAA–aspartate levels in relation to rooting. They suggested that juvenile and mature cuttings differed in auxin transport routes and in auxin conjugation; however, in their system there was no difference in adventitious root formation between different ages (Osterc and Stampar, 2011). This may be because girdling also influenced transport of sugars, which are also important for adventitious root formation and may interact with plant hormone signalling (Druege, 2009). Despite this, combined with the results here it suggests there is a high inactivation of free auxin in the cutting base of mature cuttings, which may be part of the impedance to adventitious root formation. However these findings do not rule out the possibility that another signal is also involved in regulating adventitious root formation. Further research on the mobility of the signal and on the conjugation and metabolism of auxin is required to better understand these dynamics.

### Cytokinins show different patterns over time and with age

Cytokinins also regulate adventitious root formation in a stage-specific manner and an early decrease in cytokinins is thought to contribute to the induction of adventitious root formation when combined with high auxin levels (Blakesley, 1994; Da Costa *et al.*, 2013). In the system described here only the young cuttings revealed an early peak in IAA/CK ratio (Supplementary Fig. S7) (except for IPR; Fig. 7), which confirms the theory that both a rise in auxin and cytokinin depletion control induction of adventitious roots in cuttings (Da Costa *et al.*, 2013).

In addition to concentrations, ratios of particular cytokinins have also been shown to vary with age. The iPR-type:Z-type cytokinin ratio of axillary and terminal buds decreases with maturation in *Pinus radiata* (Valdes *et al.*, 2002; Valdes *et al.*, 2004*a*). In addition, serial grafting, which reinvigorated the trees resulted in an increase in the iPR:Z ratio with the decrease in apparent maturity (Valdes *et al.*, 2003). In the pea system in this study, this decrease in IPR:Z ratio with age also holds but the change was not as marked as for pine, possibly because this change may not apply to all species as it was not found to correlate with maturation in *Pinus pinea* (Valdes *et al.*, 2004*b*).

**Fig. 10.**
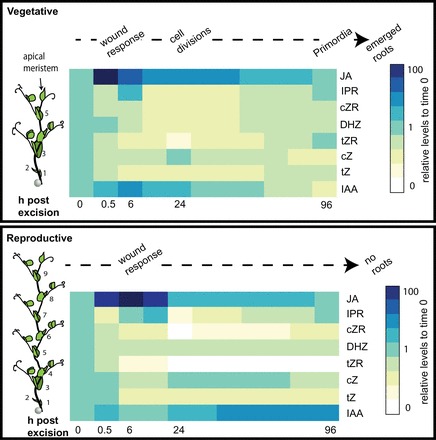
Trends in hormonal changes over time in cuttings from vegetative and reproductive plants. Schematic on the left illustrates the type and position of the cutting (black arrow). Vegetative cuttings (top) undergo wound response (JA peak) by 0.5h, cell divisions by 24h, and visible primordia by 96h. Reproductive cuttings (lower) undergo a later wound response (JA peak) at 6h, and fail to produce adventitious roots. Levels were measured at 0, 0.5, 6, 24, and 96h. The trends in between were calculated from regression lines and are meant only as a guide and not as definitive values for the time points not measured.

To summarize the differences in the time courses of hormone levels between cuttings from vegetative or floral plants, the relative changes following the excision of cuttings have been combined in Fig. 10. From these two heat maps the changes in hormones from vegetative (top panel) and reproductive (lower panel) cuttings can be clearly observed side by side and compared with the cellular stages that correspond to each time point. For example the peak in JA (dark blue square, 0.5h) in the young cuttings is delayed to 6h in the reproductive cuttings. Also the early peak in IAA (lowest row of squares) at 6h (immediately preceding cell divisions) was not present in the reproductive cuttings that do not form roots.

## Conclusions

Here, it is demonstrated that the decline in adventitious root formation is linked to the transition from vegetative to floral ontogenetic switch possibly owing to changed auxin homeostasis. Levels of jasmonic acid were also different between the young and old cutting bases but as jasmonic acid treatments did not restore adventitious rooting in the older cuttings, it is likely this is not the only signal controlling the decline in rooting. In addition, it is shown that it is unlikely that strigolactones are the cause for the age-related decline in adventitious root formation. Analysis of auxin metabolism and further manipulation of the auxin peak together with changes in cytokinins is still necessary to unravel the mechanisms controlling the decline in adventitious root formation across the ontogenetic switch.

## Supplementary data

Supplementary data are available at *JXB* online

Table S1. Features of each experiment and cutting variation.

Table S2. Collision energy settings for cytokinin analysis.

Figure S1. Diagram of grafting combinations

Figure S2. Cuttings taken within the vegetative phase increase adventitious root formation with increasing chronological age of the base and increasing height of the cutting.

Figure S3. Adventitious root formation is inhibited in cuttings from a 30-day-old plant, taken above node 2.

Figure S4. Cuttings taken during the reproductive ontogenetic phase produce less adventitious roots.

Figure S5. Grafting combinations demonstrate that the rooting percentage is dependent on the age of the root stock and hence base of the cutting.

Figure S6. Number of leaves expanded in cuttings taken at different chronological ages, or physiological ages, across different ontogenetic ages.

Figure S7. Auxin:Cytokinin ratios show an early peak in the bases of young cuttings which is not present in older cuttings.

Figure S8. JA:IAA ratios show an early peak in the bases of old cuttings which is smaller and shorter in young cuttings.

Supplementary Data

## Abbreviations:

JA: jasmonic acid
IAA: indole-3-acetic acid
CK: cytokinin
MeJA: methyl jasmonate.
