# Supplementary material for: Adventitious rooting declines with the vegetative to reproductive switch and involves a changed auxin homeostasis
Source: J Exp Bot. 2014 Dec 24;66(5):1437–52. doi: 10.1093/jxb/eru499 (PMC4339602; doi:10.1093/jxb/eru499)
Supplement: Supplementary Data [file supp_66_5_1437__index.html]

Adventitious rooting declines with the vegetative to reproductive switch and involves a changed auxin homeostasis — Adventitious rooting declines with the vegetative to reproductive switch and involves a changed auxin homeostasis — Supplementary Data 

# Adventitious rooting declines with the vegetative to reproductive switch and involves a changed auxin homeostasis

## Supplementary Data

Data files

**Files in this Data Supplement:**

- Supplementary Data - Supplementary Data
