# Supplementary material for: Adventitious rooting declines with the vegetative to reproductive switch and involves a changed auxin homeostasis
Source: J Exp Bot. 2014 Dec 24;66(5):1437–52. doi: 10.1093/jxb/eru499 (PMC4339602; doi:10.1093/jxb/eru499)
Supplement: Supplementary Data [file supp_eru499_jexbot133744_file001.pdf]

Supplementary Table S1: Features of each experiment and cutting variation.

	**Figure 3A, 4, 5, 6, 7, S2, S4, S5A,B S5E,F**	**Figure S1**	**Figure 3D, S5C**	**Figure S3**	**Figure 3G, S5D**	**Not shown**	**Not shown**	**Not shown**
**Node of cutting**	Node 2 Intact	Node 2 Intact	Node 2 Decap	Node 2 Decap	Top 2 nodes Intact	Top 2 nodes Intact	Increasing height nodes	Increasing height nodes
**Chronological age of cutting location**	Different	Different	Different	Different	Same	Same	Different	Different
**Physiological age of cutting location**	Same	Same	Same	Same	Same	Same	Different	Different
**Ontogenetic phase**	Across Veg/ floral transition	Veg	Across Veg/ floral transition	Floral	Across Veg/ floral transition	Floral	Veg	Floral
**Cutting size**	Different	different	Same	Same	Same	Same	Different (intact) /same (decap)	Different (intact) /same (decap)
Rooting trend with age	Declines	Increases	Declines	Declines	Declines	Declines		

Supplementary Table S2: Collision energy settings for cytokinin analysis.

Compound	Retention time (min)	Precursor ion	Product ion	Dwell time (sec)	Collision energy	polarity
**Transzeatin**	1.03	220	136	40	9	positive
**Transzeatin**		220	119	40	33	positive
**Ciszeatin**	1.10	220	136	40	9	positive
**Ciszeatin**		220	119	40	33	positive
**Transzeatin riboside**	2.96	352.2	220	200	9	positive
**Transzeatin riboside**		352.2	136	200	29	positive
**Dihydroxyzeatin riboside**	3.12	354.2	222	40	17	positive
**Dihydroxyzeatin riboside**		354.2	136	40	33	positive
**Ciszeatin riboside**	3.66	352.2	220	200	9	positive
**Ciszeatin riboside**		352.2	136	200	29	positive
**[15N4]-Ciszeatin**	1.10	224.2	142	40	9	positive
**[15N4]-Ciszeatin**		224.2	123	40	33	positive
**[2H5]-Transzeatin riboside**	2.96	357.2	225	200	9	positive
**[2H5]-Transzeatin riboside**		357.2	141	200	29	positive

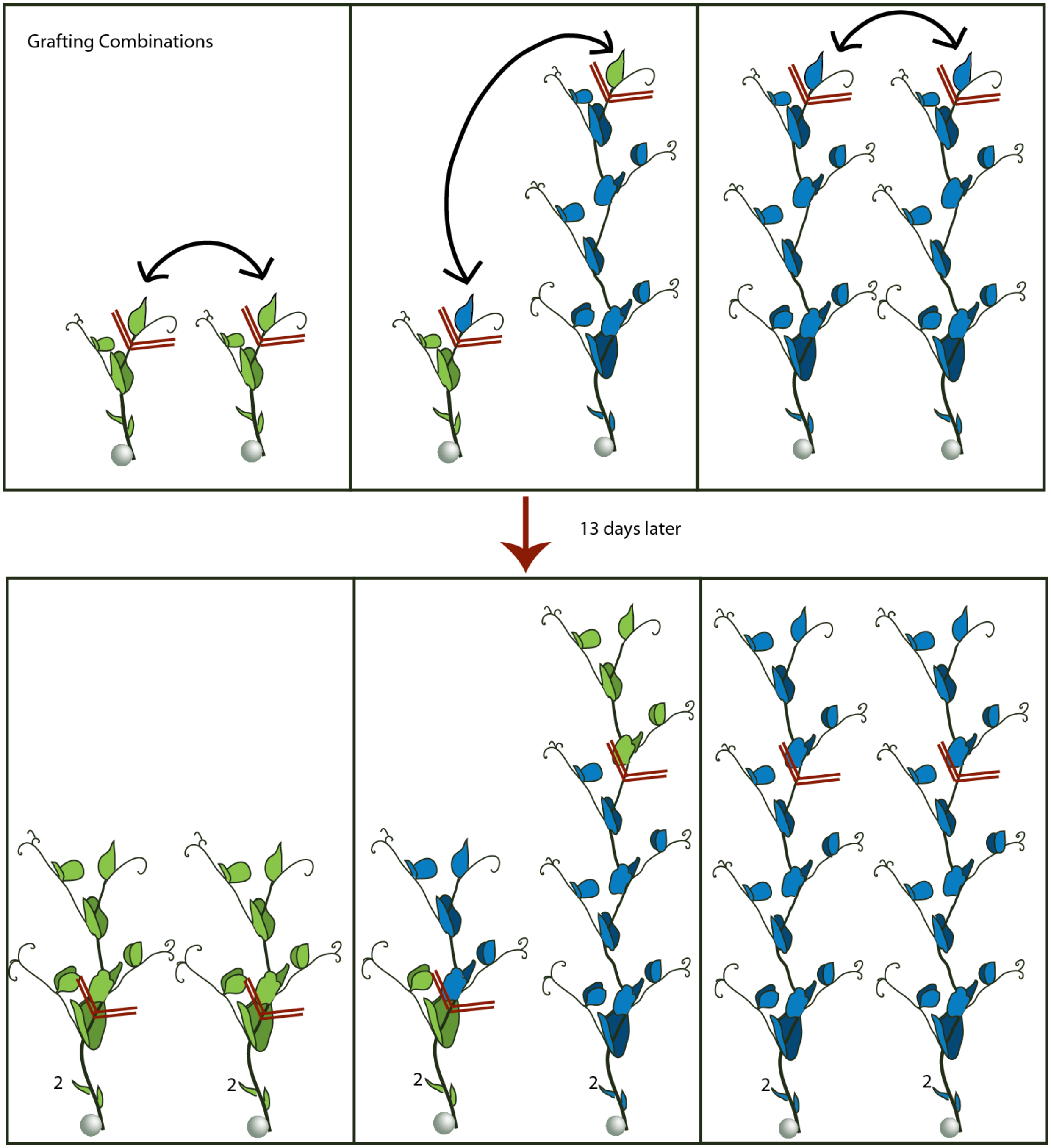

Supplementary Figure S1: Grafting combinations. Apical nodes of juvenile (green) and mature (blue) pea plants and replaced either with juvenile or mature apical meristems. 13 days later cuttings were taken above node two marked in the lower part of the figure with ‘2’.

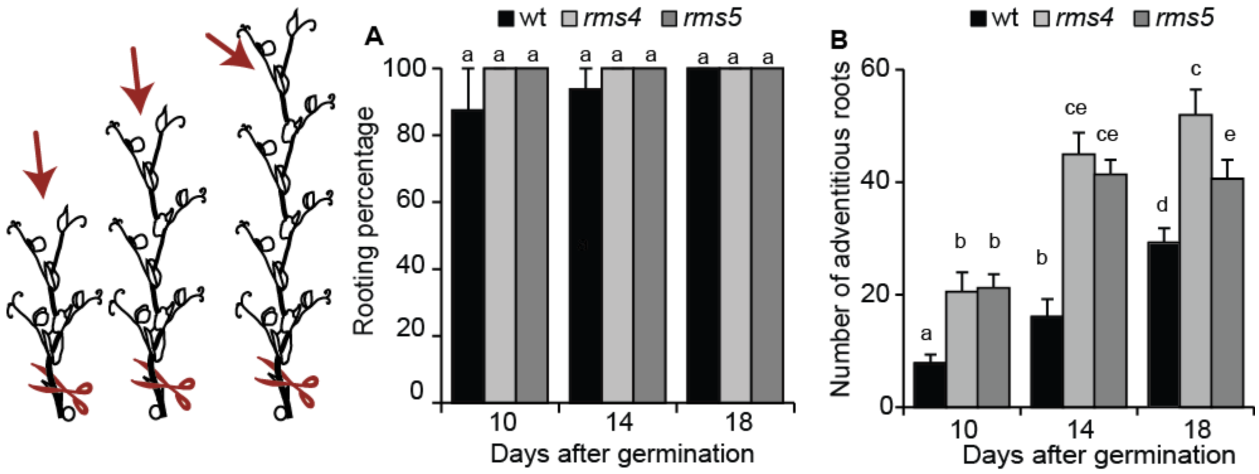

Supplementary Figure S2: Cuttings taken within the vegetative phase increase adventitious root formation with increasing chronological age of the base and increasing height of the cutting. Cuttings were taken above node two and have the same chronological age at the cutting base but different physiological age of the apical meristem and different size cuttings crossing the vegetative-floral ontogenetic switch. A) rooting percentage, B) number of adventitious roots. Means are presented ± Standard error. Different letters represent significantly different means (Students *t*test)

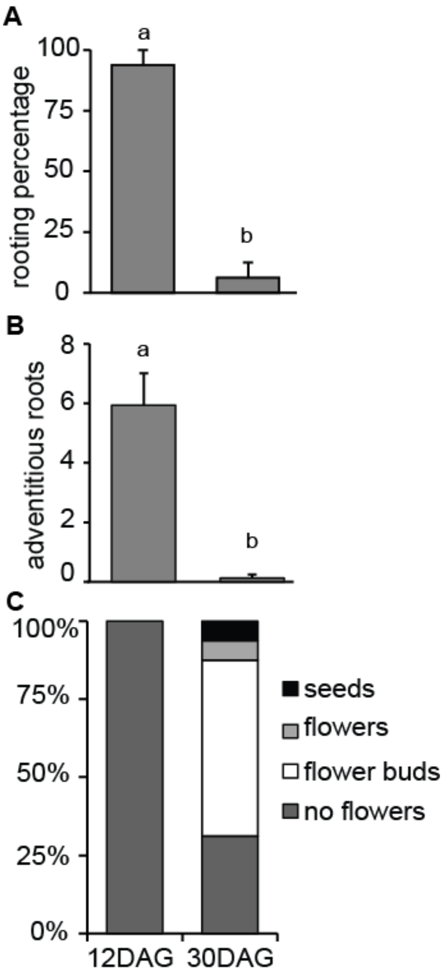

Supplementary Figure S3: Adventitious root formation is inhibited in cuttings from 30 day old plant, taken above node 2. A) rooting percentage and B) number of adventitious roots were reduced from 12 days after germination (12 DAG) to 30 days after germination (30 DAG). C) the percentage of cuttings at the end of the experiment with seeds, flowers, flower buds or no indication of flowering. Plants from this experiment were also harvested for hormone analysis presented in Figure 8, 9 and Supplementary Figure 5 and 6. Means are presented ± Standard error. Different letters represent significantly different means (Students *t*test)

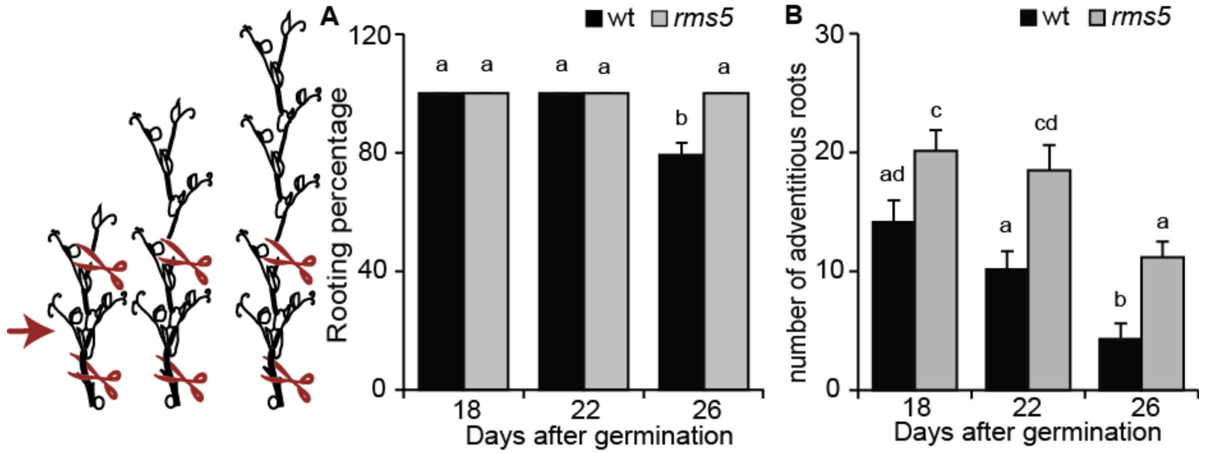

Supplementary Figure S4: Cuttings taken during the reproductive ontogenetic phase produce less adventitious roots. Cuttings were taken above node 2 but decapitated and have different chronological age at the cutting base, different physiological age of the apical meristem (before removal) and have the same size cuttings. A) rooting percentage and B) adventitious root formation declines in cuttings taken as illustrated on the left. Means are presented ± Standard error. Different letters represent significantly different means (Students *t*test).

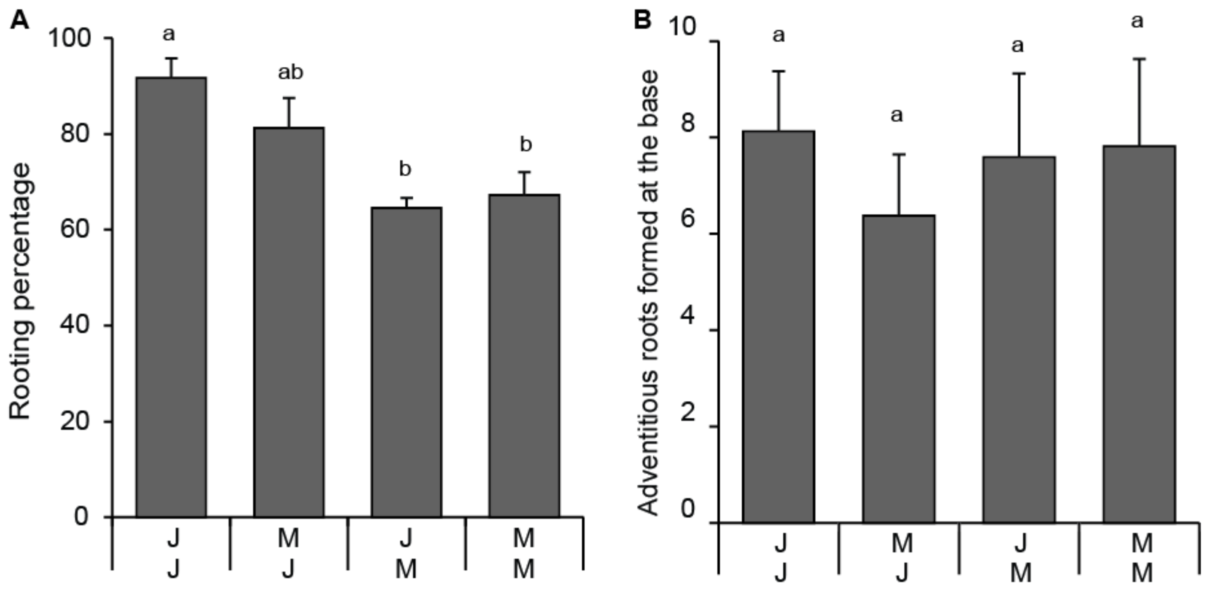

Supplementary Figure S5: Grafting combinations demonstrate that the rooting percentage is dependent on the age of the root stock and hence base of the cutting. J/J are juvenile shoots grafted onto juvenile roots; M/J are mature shoots grafted onto juvenile roots; J/M are juvenile shoots grafted onto mature roots; M/M are mature shoots grafted onto mature roots. A) Rooting percentage. B) Number of adventitious roots. A,B) Means are presented ± standard error; n = 16. Different letters represent significantly different means (Students *t*test)

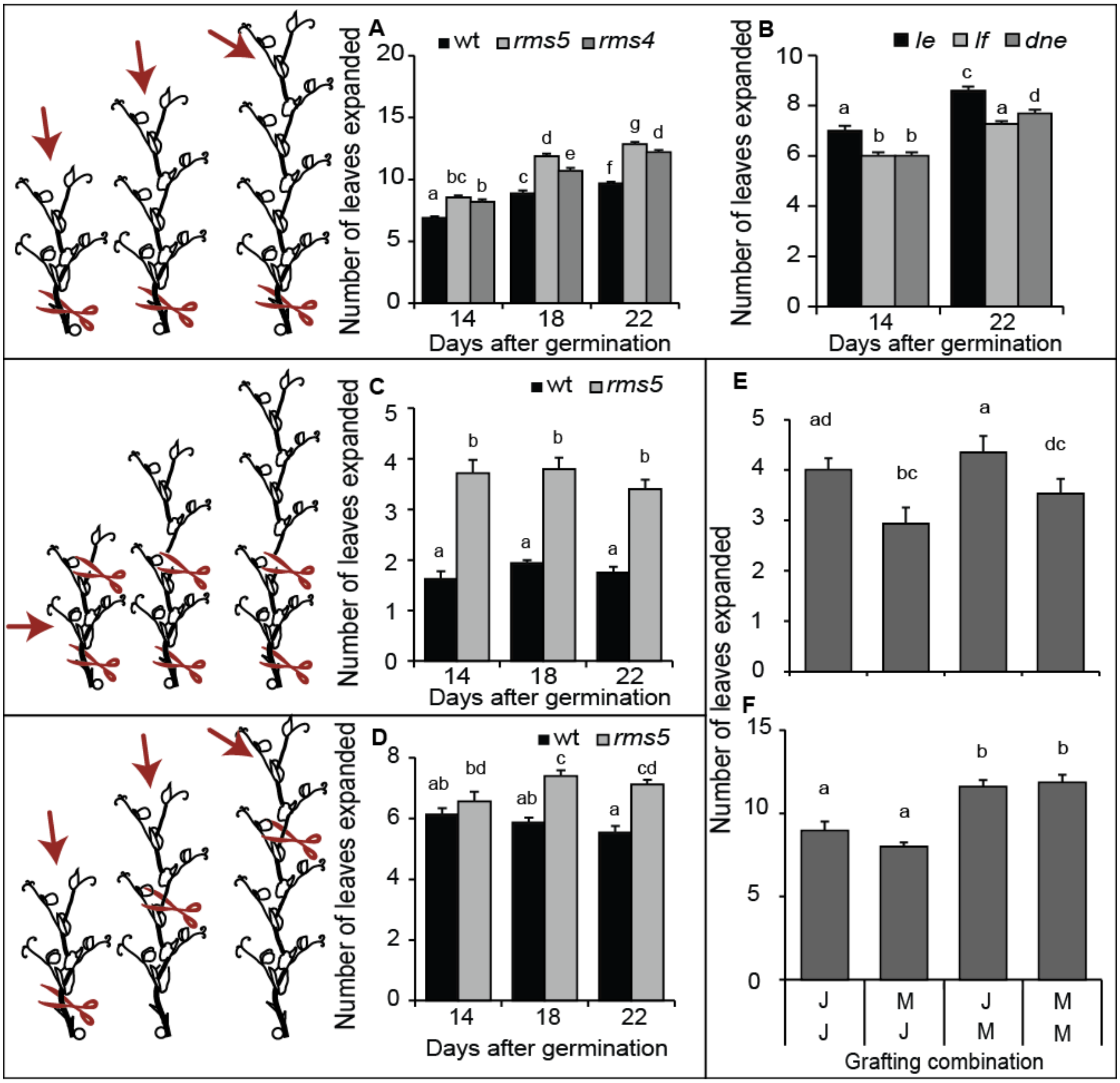

Supplementary Figure S6: Number of leaves expanded in cuttings taken at different chronological ages, or physiological ages, across different ontogenetic ages. A,B,C,D,) Changes in number of leaves expanded with age in cuttings taken as illustrated on left and in Figure 2A, C and E respectively. A,C,D) are in wildtype and strigolactone mutants (*rms5*, *rms4*), B) *le* control; *dne* and *lf* early flowering mutants; E,F) Number of leaves expanded above the graft in Torsdag (E) and W6 22593 (F) graft combinations. Means are presented ± Standard error. Different letters represent significantly different means (Students *t*test).

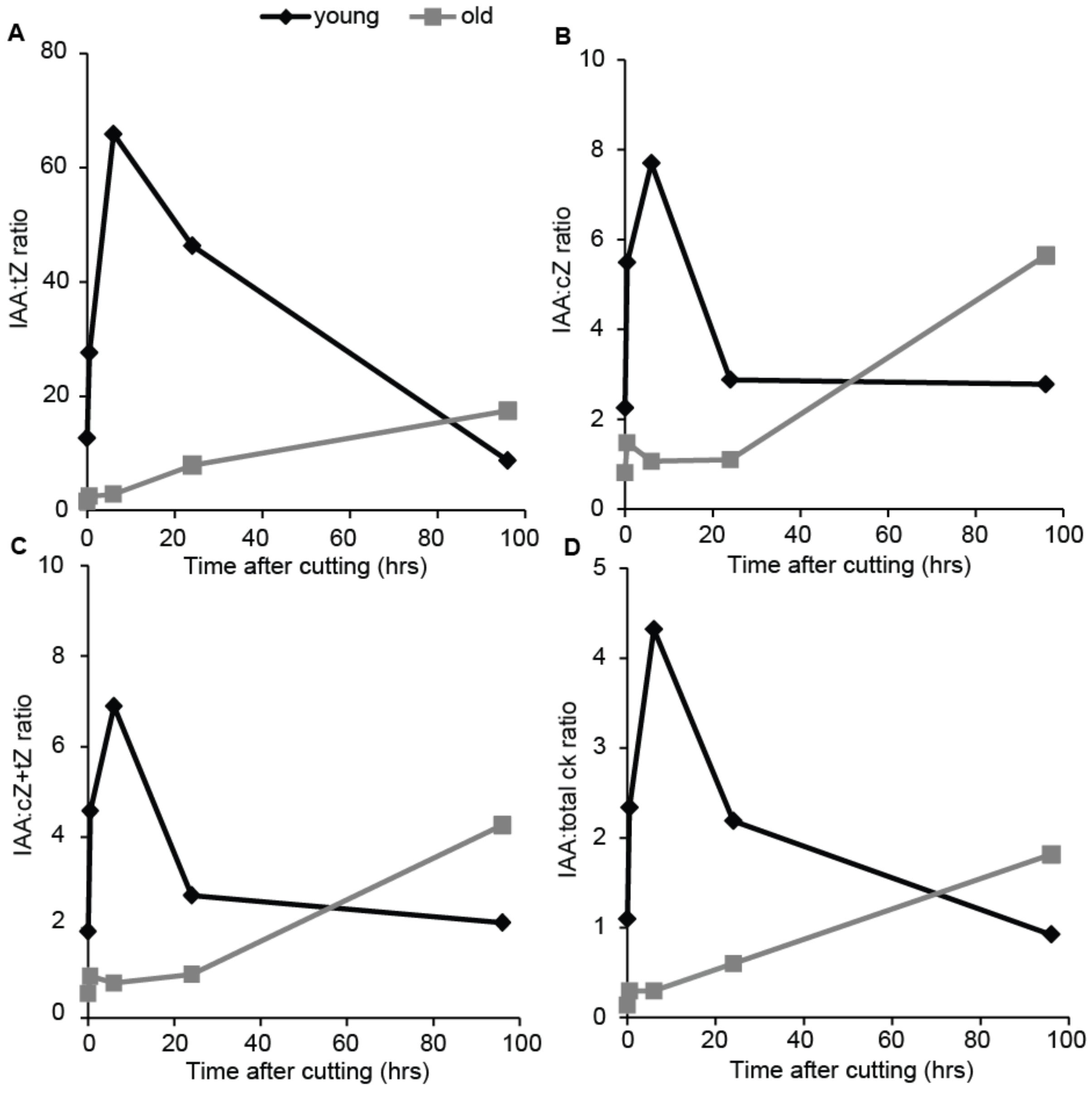

Supplementary Figure S7: Auxin:Cytokinin ratios show an early peak in the bases of young cuttings which is not present in older cuttings. A) IAA:transzeatin, B) IAA:Ciszeatin, C) IAA:cisZeatin +transZeatin , D) IAA:total cytokinin at 0, 0.5, 6 , 24 and 96 hours after cutting.

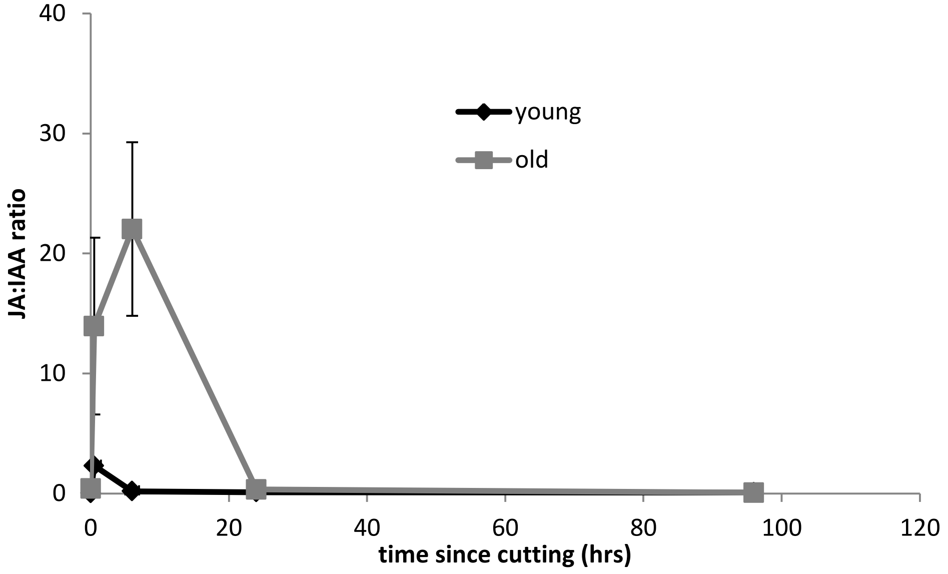

Supplementary Figure S8: JA:IAA ratios show an early peak in the bases of old cuttings which is smaller and shorter in young cuttings. Means presented ± standard error bars.
